# Value Sensitive Design to Achieve the UN SDGs with AI: A Case of Elderly Care Robots

**DOI:** 10.1007/s11023-021-09561-y

**Published:** 2021-05-31

**Authors:** Steven Umbrello, Marianna Capasso, Maurizio Balistreri, Alberto Pirni, Federica Merenda

**Affiliations:** 1 Institute for Ethics and Emerging Technologies, University of Turin, Via Sant’Ottavio, 20, 10124 Turin, TO Italy; 2 Scuola Superiore Sant’Anna, Piazza Martiri della Libertà, 33, 56127 Pisa, Italy; 3 University of Turin, Via Sant’Ottavio, 20, 10124 Turin, Italy

**Keywords:** Care robots, Value sensitive design, Autonomous systems, AI ethics, Applied ethics

## Abstract

Healthcare is becoming increasingly automated with the development and deployment of care robots. There are many benefits to care robots but they also pose many challenging ethical issues. This paper takes care robots for the elderly as the subject of analysis, building on previous literature in the domain of the ethics and design of care robots. Using the value sensitive design (VSD) approach to technology design, this paper extends its application to care robots by integrating the values of care, values that are specific to AI, and higher-scale values such as the United Nations Sustainable Development Goals (SDGs). The ethical issues specific to care robots for the elderly are discussed at length alongside examples of specific design requirements that work to ameliorate these ethical concerns.

## Introduction

The virus SARS-CoV-2 has uncovered fundamental inequalities in medical, social, economic and political domains across the globe. The practice of care, that is, caring for patients—both ill with the COVID19 disease and those living in isolation from it—has been particularly challenging for caregivers and care-receivers since there is no apparent solutions. The village of Eraviperoor in India has recently deployed a series of care robots that are equipped to provide patients in their local medical center with medicines, bedsheets, and food, weighing up to 8 kg (Kuttoor, 2020). In the UK, researchers studying assisted living at Heriot-Watt University in Edinburgh are employing co-design approaches to develop care robots to combat COVID care isolation (Macdonald, 2020).

These recent examples are part of a larger trend in the automation and deployment of information and communication technologies (ICTs), and increasing use of robotics within the domain of care (Mordoch et al., 2013). Consequently, the diaspora of care robots globally raises ethical, social, cultural and political concerns. Three pressing issues that have emerged in the deployment of robots are: the reliability of care robots to provide beneficial aid, their medical fidelity to ensure proper treatment, and whether they can provide sufficient companionship and comfort to those who are isolated. To confront these issues, a significant body of literature has emerged to determine how to integrate applied ethical approaches towards the design and deployment of these types of autonomous systems to ensure beneficial ends (e.g., van Wynsberghe, 2012, 2013a, 2013b, 2015). Intervening at the design phase has been a long-standing position in the field of responsible innovation and has recently been the focus of various multinational governance and funding bodies (United Nations, 2019; van den Hoven & Jacob, 2013; van Lente et al., 2017).

By focusing primarily on care robots for the elderly, this paper aims to provide a conceptual investigation of the ethical issues and human values that emerge within the framework of value sensitive design (VSD), and a principled approach to the design of technologies *for* human values (Friedman & Hendry, 2019). In doing so, this paper builds on the Care Centered VSD (CCVSD) approach advocated by van Wynsberghe (2013a, 2013b), to expand VSD to include other sources of values such as: the United Nations Sustainable Development Goals (SDGs), ethics guidelines for trustworthy AI by the High-Level Expert Group on Artificial Intelligence (HLEG AI) (High-Level Expert Group on AI, 2019), and ‘norms’ that are specifically related to beneficial autonomous systems design, such as the AI for Social Good (AI4SG) principles by Floridi et al. (2020). By adopting the multi-tiered approach to AI design via VSD proposed by Umbrello and van de Poel (2021), this paper provides a thorough analysis of care robot design for the elderly, which more accurate maps onto the ethics of care proposed by van Wynsberghe (2013a, 2013b).

Previous studies have focused solely on the ethical issues of care robots (Sharkey & Sharkey, 2012; Vandemeulebroucke et al., 2018) and the use of care ethics in their design (van Wynsberghe, 2016). This paper is different from these in its in its multi-tiered approach to implementing VSD which draws on multiple sources of values at the domain, technological, and international levels as they pertain to autonomous systems. We do so by distinguishing between values to be promoted (as much as possible) [i.e., the UN SDGs], values to be respected (as much as possible) [i.e., HLEG AI] as well as contextual values such as those derived from stakeholder elicitation (i.e., elderly patients using care robots). This will contribute to the salient design of these types of artificial intelligence systems at a global level.

This paper is divided into the following parts. Section 2 reviews existing literature on VSD, and outlines the basic methodology that will be employed throughout the paper. Section 3 discusses the current state-of-the-art care robots with regard to ethics and the design of care robotics, particularly focusing on the work of van Wynsberghe. Section 4 introduces the multi-tiered VSD approach of Umbrello and van de Poel (2021) and outlines ways to apply this approach to elderly care robots. Section 5 looks in greater depth at the ethical issues that are particular to elderly care robots and how the proposed approach can be employed to provide key design requirements to meet those challenges. Section 6 presents our conclusions.

## The Value Sensitive Design Approach

Value-sensitive design (VSD) is defined as “a theoretically grounded approach to the design of technology that accounts for human values in a principled and comprehensive manner throughout the design process” (Friedman et al., 2013, p. 2). Opposing the neutrality thesis, according to which technological systems are neutral tools that depend on the users for their status, VSD promotes an interactional understanding of technological systems. An interactional understanding implies that the impact of technological systems on users is shaped by the features of their design, the context in which they are used, and the people involved in their use (van den Hoven et al., 2015). Therefore, the main theoretical aim of VSD is to incorporate an investigation on moral and social values and a clear and coherent methodology into the overall design and implementation process of systems.

**Fig. 1:**
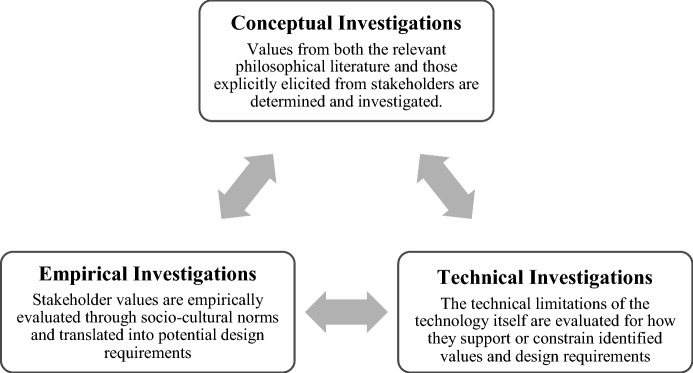
The recursive VSD tripartite framework employed in this study. *Source* Umbrello (2020a)

In the VSD literature, the definition of value given by Friedman et al. refers to ‘‘what a person or group of people consider important in life’’(Friedman et al., 2013, p. 2). Thus, the identification of values in VSD varies depending on the specific systems, contexts, stakeholders, and application domain under analysis. Values, however, should not be construed as mere preferences, although preferences are an important factor in design decisions. There has been debate on the philosophical foundations of values in VSD (Le Dantec et al., 2009; Manders-Huits, 2011). More recently, Friedman et al. have grounded the moral values, regardless of their situational expression, in the following universal values: *human well-being*, *justice,* and *dignity* (Friedman & Hendry, 2019; Umbrello, 2020b). Originally, VSD was developed in the information and communication technology domain (Friedman & Kahn Jr., 2003; van den Hoven, 2007), but the approach is now more widely adopted and extends into different domains, sometimes under the alternative heading of Design for Values (van den Hoven et al., 2015). However, the methodology, which is based on the tripartite methodology of Friedman et al. (2002), remains the same. It is composed of three types of iterative and integrative investigations: conceptual, empirical and technical (Fig. 1).

The conceptual investigation involves two primary activities: the identification of stakeholders that are or will be affected by the system, and the identification and definition of values and possible trade-offs. The empirical investigation examines stakeholders’ “understandings, contexts, and experiences” (Friedman & Kahn Jr., 2002, 2003). Finally, the technical investigation is concerned with the specific features/architecture of new or existing systems, and specifically in terms of how they can support or constrain the implementation of values. Since its inception, VSD has accompanied the investigation of values by a range of social science methods. Recently, Friedman and Hendry (2019) have proposed 14 more specific methods that can be used: (1) stakeholder analysis; (2) designer/stakeholder explicitly supported values; (3) coevolution of technology and social structure; (4) value scenarios; (5) value sketches; (6) value-oriented semi-structured interview; (7) granular assessments of magnitude, scale, and proximity; (8) value-oriented coding manual; (9) value-oriented mock-ups, prototypes, and field deployments; (10) ethnography focused on values and technology; (11) model for informed consent online; (12) value dams and flows; (13) value sensitive action reflection model; and (14) envisioning cards.

**Fig. 2:**
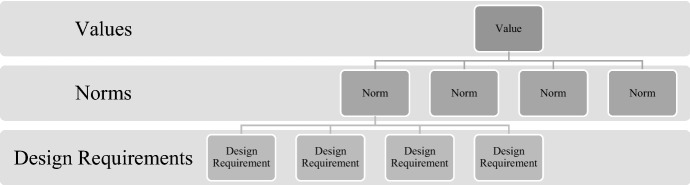
Values hierarchy. *Source* van de Poel (2013)

Tools such as the ‘values hierarchy’ developed by van de Poel (2013) are crucial in translating values into more tangible design requirements (Fig. 2). A values hierarchy is a structure that is comprised of three basic layers: (1) values, which are general values that need to be promoted for their own sake; (2) norms, which are restrictions on or prescriptions for action, and (3) design requirements, which are a set of specific criteria that should be achieved as much as possible. The hierarchies can be constructed top-down as well as bottom-up. The former through the means of a non-deductive and context-dependent specification of high-level elements into lower ones and the latter relating lower-level elements to higher ones, on the basis of ‘for the sake of’—that is, in such a way in which the higher has a motivating and justifying role for the lower.

Despite its beneficial applications, VSD has received criticism due to the fact that it does not explicitly recommend or forbid commitment to a particular ethical theory (van den Hoven et al., 2015). Critics argue that VSD lacks a clear and normative methodology to distinguish general moral values from mere stakeholders’ preferences (Albrechtslund, 2007; Manders-Huits, 2011). For example, bottom-up approaches that argue that it is better to elicit values from stakeholders often lack the ability to justify value prioritizations or value trade-offs normatively (Borning & Muller, 2012; Le Dantec et al., 2009). Moreover, the focus on stakeholders’ preferences may lead to the exclusion of other values and actors that are of ethical importance in VSD. Other approaches provide a list of values, as in the case of Friedman et al. (2017) that propose a list of 14 values related to the design of information systems. However, these top-down approaches are often too indeterminate to assess specific contexts and systems critically.

## Care Robots and Care Centered VSD

The VSD approach to addressing issues of design shows how the evaluation of systems cannot be disengaged from their role and tasks that they are expected to fulfil. This is a fundamental aspect of the health care domain, where systems can shape the decision-making processes, practices, and behaviours of vulnerable persons. One definition of care robots is: “Carebots are robots designed for use in home, hospital, or other settings to assist in, support, or provide care for the sick, disabled, young, elderly or otherwise vulnerable persons” (Vallor, 2011, p. 252). The literature on robot ethics, and care robots in particular, focus on two perspectives: the autonomy and vulnerability of patients as care-receivers (Pirni et al., 2017; Sharkey & Sharkey, 2012; Sorell & Draper, 2014; Sparrow & Sparrow, 2006); and on caregivers and standards of care (Sharkey, 2014; Vallor, 2011). While these contributions rightly claim that the introduction of robots has inevitably changed healthcare practices, it is only very recently that the necessity of a normative framework coupled with a design-oriented perspective is recognized by the works of van Wynsberghe on Care Centred Value Sensitive Design (CCVSD).

CCVSD has been developed in response to the ethical issues surrounding the use of care robots and the criticisms against VSD outlined in the previous section. Van Wynsberghe has promoted the CCVSD approach by demonstrating how it may be used with a twofold aim in mind. First, as a new framework specifically tailored to evaluate care robots and practices based on definite standards, which are not currently provided by the International Organization for Standardization (ISO 2011). Second, as a means to overcome the lack of a transparent and explicit normative grounding in VSD (van Wynsberghe, 2012, 2013a, 2013b, 2016).

According to van Wynsberghe, a comprehensive evaluation of care robots and practices can be accomplished by the integration of the traditional VSD approach with normative criteria and elements from a care ethics perspective. This helps to identify and establish which values should be promoted in the VSD process (van Wynsberghe, 2013a). Following the care ethicist Tronto (1993), van Wynsberghe identifies four fundamental value of care to be promoted in the design of systems: (1) attentiveness, or the caregiver’s capacity to recognise the needs of the care-receiver; (2) responsibility, or the caregiver’s concern with meeting the needs of the care-receiver; (3) competence, which means the caregiver’s capacity to execute an action in order to fulfil the needs of the care-receiver; and (4) responsiveness or reciprocity, which is the care-receiver’s capacity to guide the caregiver and the instauration of a reciprocal interaction (van Wynsberghe, 2012, 2013a, 2013b, 2016).

**Fig. 3:**
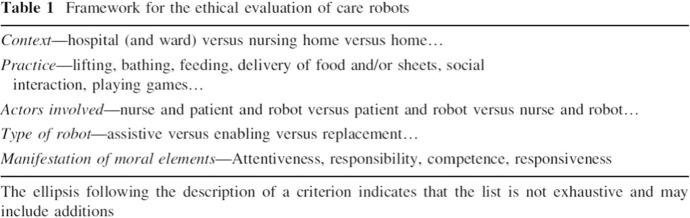
*Care-Centered Framework*. *Source* van Wynsberghe (2013a, p. 420)

Van Wynsberghe argues that these four elements are crucial in any care practice that impacts on both caregivers and care-receivers, due to the ethical importance of the relationship dynamic, i.e. the distribution of roles and responsibilities between them (van Wynsberghe, 2013a). Van Wynsberghe’s approach is intended to help ethicists and designers in their investigations with the use of two frameworks: (1) a care-centred framework, which consists of five components that require attention in a care analysis: context, practice, actors, type of robot, manifestation of the four moral elements (Fig. 3); (2) a specific CCVSD methodology framework, which proactively guides ethicists and designers from data collection, value analysis of the care practices (with and without the robot), and of the robot’s capabilities, to scenario comparison and recommendation for design.

CCVSD relies on the concept that care practice is a response to the needs of ‘the other’ (van Wynsberghe, 2016) determining the four values to be included in the design of systems. However, if this methodological approach has the merit of emphasizing the importance of a normative evaluation in VSD, it dismisses other potentially relevant values that may play a pivotal role in care practices. Other approaches have begun to expand and develop CCVSD further. For example, Santoni de Sio and van Wynsberghe (2016) rely on a nature-of-activities approach for a CCVSD of care robots. By providing a clearer philosophical foundation and more detailed descriptions of the nature of care activities, this collaboration extends the CCVSD approach by leaving space for the inclusion of other values and other philosophical traditions, such as individual autonomy.

Van Wynsberghe recognises that the list of values in the CC framework is not exhaustive and allows for additions (Fig. 3), although her deliberate exclusion of an ellipsis for the ‘moral elements’ (Fig. 3) indicates that she does not leave this value open for the inclusion of other values. In her recent work she acknowledges that in a possible future scenario, a care robot that is embedded with a sufficient level of AI would be able to engage in a degree of attentiveness and competence in such a way as to be conceived as a ‘reciprocal partner’ for the care-receiver (van Wynsberghe, 2016). This scenario may lead to the creation of an unprecedented and new care practice that would be impossible to compare with existing practices (van Wynsberghe, 2016). However, this leaves unexplored the most interesting implications of the impacts that such care robots may have on care practices. It also fails to consider what these impacts would entail for the approach of CCVSD. According to van Wynsberghe, CCVSD can still be used in these cases, but she relegates this line of inquiry to future research (van Wynsberghe, 2016).

However, health systems are now using a variety of care robots, from physical robots to embodied AI, or virtual assistants, which inevitably have a radical impact on care practices. For example, Amazon Alexa can now provide medical advice to patients, as part of a collaboration with the UK National Health Service (NHS).^1^

In the case of these new AI-driven systems the manner of ‘manifestation of moral elements’ is still dependent on the actors that contribute to the care practice. Nonetheless, the introduction and adoption of these systems requires that external criteria to the caregivers and care-receivers relationship be considered when assessing the impact on care practices in a comprehensive way. Indeed, the potential impact of AI-driven systems on care practices goes beyond considerations that are exclusively based on the traditional bilateral relationship between care-receivers and caregivers to include the health system as a whole, and the third-party providers that deploy and implement systems into care practices. Van Wynsberghe and Li (2019) recognise that the introduction of AI-driven systems may shape and transform the healthcare system, with the reallocation of resources, competences, and responsibilities of the healthcare staff. Moreover, a commitment to good care practices should consider the ramifications of third-party providers that are now entering the domain of healthcare. Big tech and market-driven companies such as Amazon may become involved in public research to further their own healthcare agenda; and this has raised concerns among scholars (Sharon, 2016, 2020). Under the umbrella term of ‘actors involved’ in a care practice, the specific type of AI-driven robot that can act as a ‘reciprocal partner’ requires a reference to the healthcare system, the health medical staff, and the type of providers (designers and companies) that implement such robots in care practices. The introduction of AI-driven robots in healthcare requires a comprehensive criteria for good care, especially if the social dimension that is inherent in care practices is to remain (Coeckelbergh, 2010).

This necessitates the consideration of values that are particular to AI systems and that can better address the broader ethical and social concerns that arise from those systems. A good starting point is the cluster of values distilled by the European Commission’s HLEG AI: *explicability*, *autonomy*, *nonmaleficence*, and *fairness*, which are used in emerging autonomous systems underlying new types of care robots, and could constitute a first step towards an VSD-AI4SG approach to AI design.

## A Multi-tiered Approach to AI Design with VSD

The preceding section outlined the CCVSD approach and what merits it has as a branch of the VSD methodology.

The CCVSD methodology that van Wynsberghe (2013b) formulated is able to adapt itself to the values of care that are associated with the domain in which care robots find themselves, and this adaptability to discrete design programs is a fundamental directive of VSD (Friedman & Hendry, 2019; Friedman & Kahn Jr., 2002; 2003). Given that the artefacts examined in this paper are of a similar nature to one another, using CCVSD as a starting point is logical. CCVSD is a useful methodology in this context because it concentrates on the impact that care robots may have on care practices and gives a normative foundation to VSD. Our primary aim is to develop the CCVSD further by providing a more nuanced normative approach. When the ‘care practice’ at stake is impacted by AI-driven systems, an evaluative and justificatory analysis arguably requires a reference not only to care ethics, but also to considerations operating on a societal level in which such practice is embedded. There might also be other actors beyond individual caregivers and care-receivers. The proposed shift to VSD-AI4SG is one way to address this issue.

By combining CCVSD with other norms and values that are specifically adapted to autonomous systems such as AI4SG norms (Floridi et al., 2020), and the values of the HLEG AI (High-Level Expert Group on AI, 2019), the design of AI systems can be made care-sensitive to avoid doing harm, and be actively directed towards social good, even beyond the deployment domain. Umbrello and van de Poel (2021) argue for the implementation of UN Sustainable Development Goals (SDGs) as a useful approximation of what can be collectively believed to be valuable societal ends (Umbrello & van de Poel, 2021, p. 1). The following subsections describe their approach, which is then deployed in Sect. 5.

### VSD for AI

Various considerations need to be taken into account when reviewing the design of AI systems. There is no longer any doubt as to whether AI systems will have significant and lasting sociocultural, economic and ethical impacts (Baum, 2016; Khakurel et al., 2018) although, many of the ethical impacts that AI systems are implicated in are not explicitly accounted for in the original value protocols that VSD scholars have proposed for other ICTs (Friedman & Kahn Jr.,^2002^; ^2003^; Umbrello, 2019). The values proposed by the HLEG AI (High-Level Expert Group on AI, 2019) provide an excellent starting point for considering values that are explicitly implicated by AI systems. Having protocols of AI specific values are useful for ensuring a certain level of top-down alignment when engaging in AI design programmes, despite the almost certain need for bottom-up stakeholder engagement and value elicitations to make AI alignment robust and holistic (Umbrello & van de Poel, 2021).

**Fig. 4:**
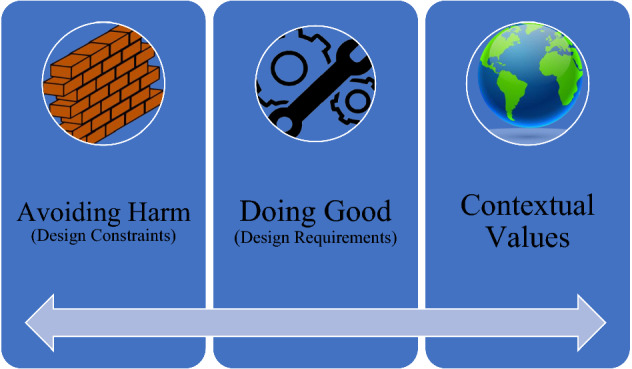
Three sources of values for VSD for AI4SG

The design of these types of AI systems needs to avoid doing harm and contribute to social good. Umbrello and van de Poel (2021) propose the UN Sustainable Development Goals (SDGs) as a larger set of values for social good to design AI systems *for* human values (discussed further in the next sub-section). The approach they propose is fundamentally predicated on three sources of values (Fig. 4): (1) avoiding harm, which should be construed as boundary conditions or design constraints, (2) doing good, which is associated with designing primarily for social good can be construed as design requirements and criteria, and (3) reflection of a specific context, that can take the form of avoiding harm and/or doing good. The three sources of value can overlap in many cases, although they require individual attention in the design process.

While the third contextual source can widely differ depending on a number of varying relevant factors, Umbrello and van de Poel (2021) argue that it is nonetheless useful to use protocols of values for (1) and (2):

- The first tier of values should be taken into account in any application of AI. To ensure that AI does not do more harm than good, they propose making use of the values articulated by the HLEG AI and translated through the more concrete AI4SG norms into technical design requirements.
- The second tier of values that they actively seek to promote is social good through AI. They propose using the SDGs as first-order operationalisations of what it means to contribute to social good through AI. Here the idea is that the SDGs, to which an AI application contributes, will be specific for that application (Umbrello & van de Poel, 2021, p. 5)

The next sub-section discusses the SDGs (tier 2) and sub-Sect. 4.3 discusses the AI4SG meanings and factors (tier 1).

### SDGs

**Fig. 5:**
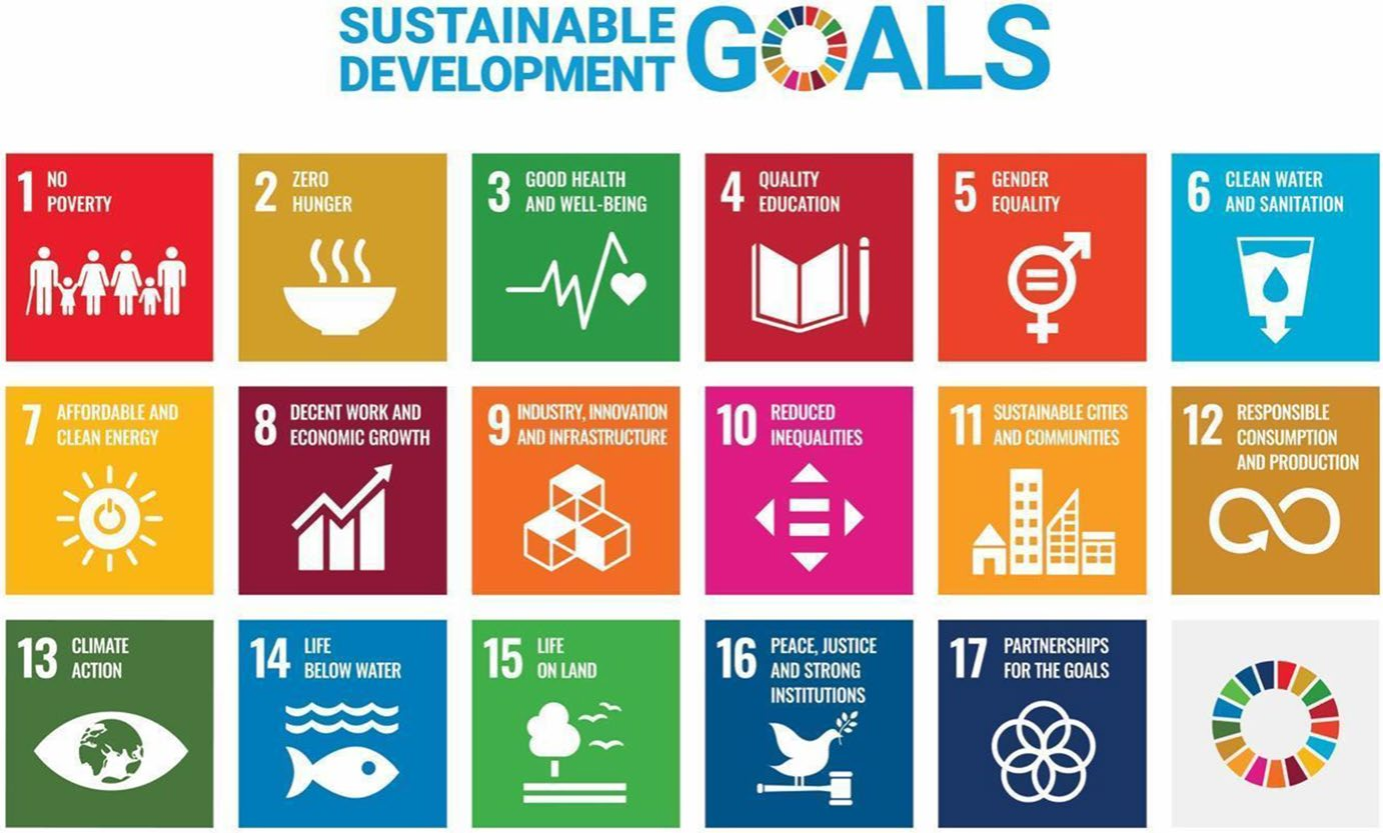
United Nations Sustainable Development Goals. *Source* United Nations (2019)

In 2018 the United Nation drafted a proposal of objectives that are to be designed and implemented for a safe and sustainable future, with the ultimate goal of global peace (United Nations, 2018). The foundation of this proposal is built on 17 actionable sustainable development goals (SDGs) (Fig. 5). The goals are presented as being necessarily combinatory and complimentary rather than hierarchically ordered or prioritized. The ultimate objective is a synergistic and symbiotic approach to achieving all of these SDGs.

The UN’s underlying philosophical approach frames technologies in an interactional way, arguing that technologies co-vary with their societal and cultural contexts, rather than being purely deterministic artefacts or instrumental tools. This institutional direction allows the SDGs to be tackled holistically rather than haphazardly, and likewise envisions technologies not only as a potential problem that exacerbates issues, but as a potential solution (Umbrello & van de Poel, 2021). Umbrello and van de Poel (2021) use the SDGs as a higher-order source of values in the VSD for AI systems (alongside others such as those used by the HLEG AI) in line with the global trend towards a set of common goals. Given that technology is a central force in the exacerbation, as well as amelioration, of the issues that the SDGs are proposed to address, they provide a useful set of higher-order guidelines to design it *for* human values.

### AI for Social Good Factors

**Fig. 6:**
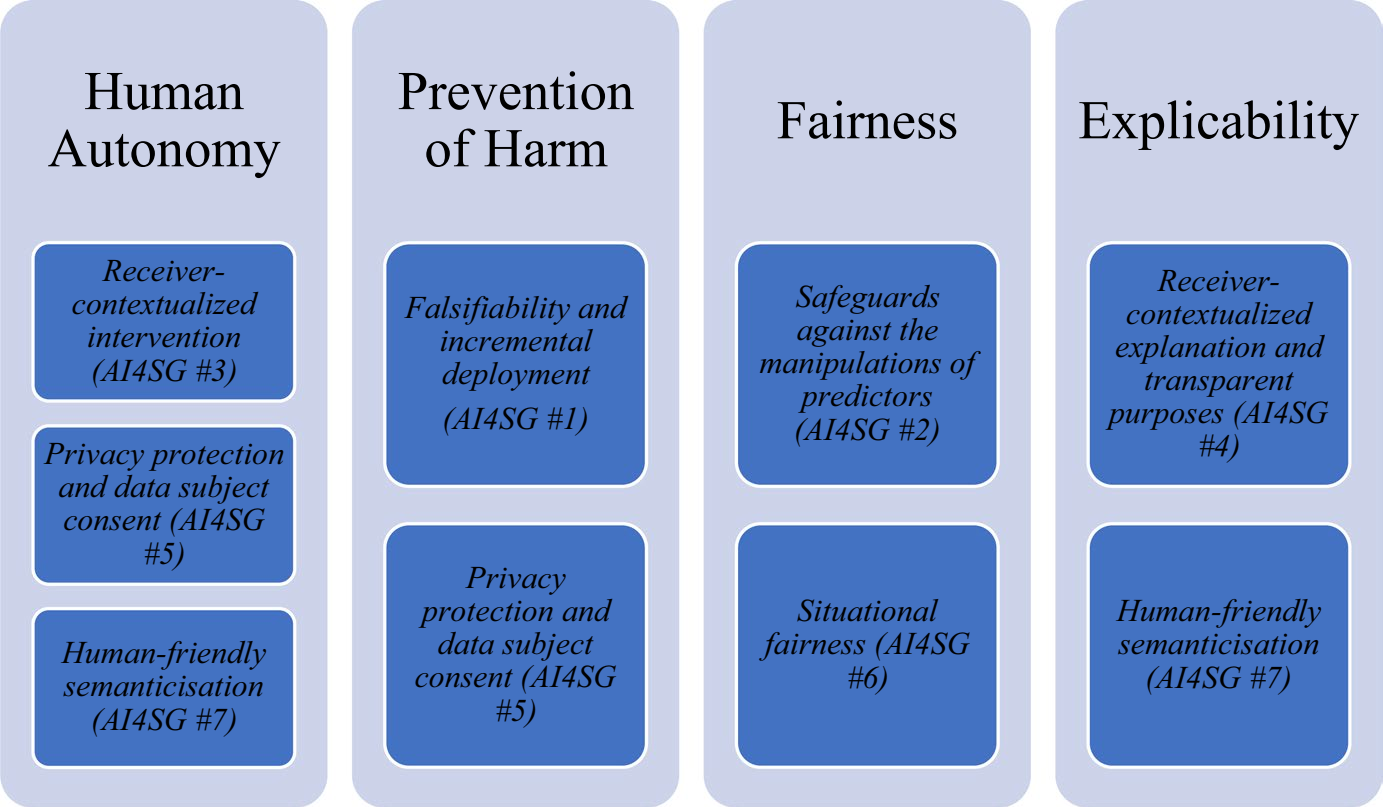
Relationship between higher-order values of the EU HLEG on AI and AI4SG norms. *Source* Umbrello and van de Poel (2021)

Umbrello and van de Poel (2021) argue that the most comprehensive and streamlined summary of the AI4SG factors are those recently produced by Floridi et al. (2020). The seven factors that are particularly relevant for the design of social good in AI are: (1) *falsifiability and incremental deployment*; (2) *safeguards against the manipulation of predictors*; (3) *receiver-contextualized intervention*; (4) *receiver-contextualized explanation and transparent purposes*; (5) *privacy protection and data subject consent*; (6) *situational fairness*; and (7) *human-friendly semanticisation* (Floridi et al., 2020, p. 3). Although these seven factors are discussed separately, like the SDGs, they are nonetheless co-dependent and co-vary with one another, making them inextricably linked in an effort to achieve AI4SG. Umbrello and van de Poel (2021) argue that the

seven factors each relate, in some way, to at least one of the four ethical principles that EU High-Level Expert Group on AI lays out: respect for human autonomy, prevention of harm, fairness and explicability. This mapping on to the more general values of ethical AI is not insignificant, any divergences from these more general values has potentially deleterious consequences. What the seven factors are meant to do then is to specify these higher-order values into more specific ‘norms’ and design requirements (Umbrello & van de Poel, 2021, p. 8) (Fig. 6).

For the purposes of brevity, this paper does not discuss the definitions of the seven factors outlined in Floridi et al. (2020). However, Umbrello and van de Poel (2021) argue that the AI4SG factors function like ‘norms’ following on from van de Poel’s (2013) characterization of norms as being framed as ‘maximizing’ or ‘minimizing’ certain value or design requirements, thus bridging the gap between abstract values (e.g., HLEG AI, UN SDGs) and concrete design requirements. This is discussed further in the next sub-section.

### AI4SG-VSD Process

**Fig. 7:**
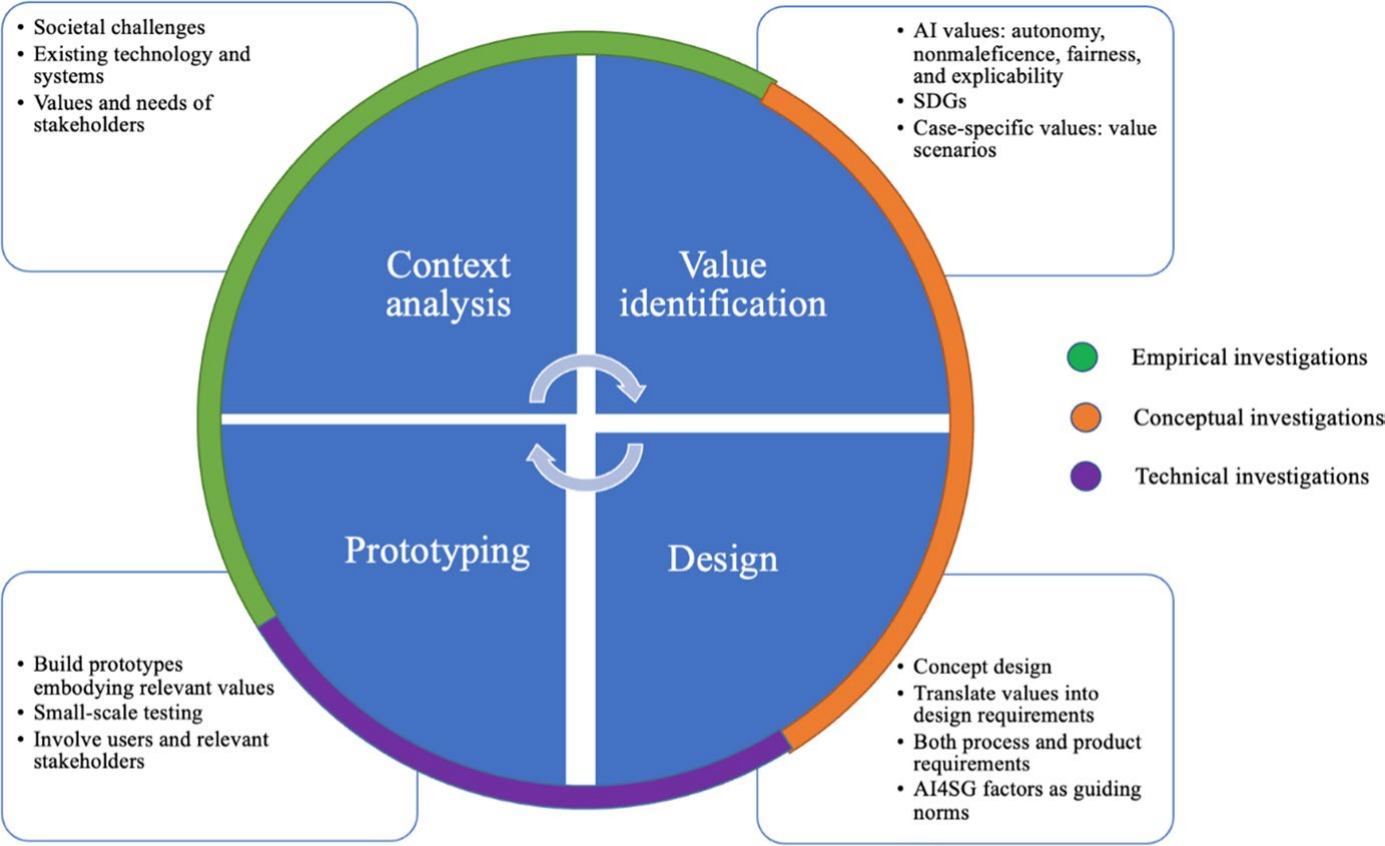
AI4SG-VSD design process.n *Source* Umbrello and van de Poel (2021)

As outlined in the Introduction, the aim of this paper is to draw from the AI4SG methodology of designing by using VSD as part of a proposal for the future of care robots for the elderly Umbrello and van de Poel (2021). In other words, the UN SDGs and HLEG AI principles are used as the aims from which more specific values can be derived for doing good, while the normative AI4SG principles are used as the basis for avoid harm. Figure 7 illustrates how engineers can initiate investigations in their design program. Although differing from one project to another, the proposed framework provides a general outline that practitioners can follow to ensure they touch on the fundamental points proposed in this framework.

The four-stage iterative process that Umbrello and van de Poel (2021) propose is composed of: (1) context, (2) value identification, (3) formulating design requirements, and (4) prototyping.

#### Context

The sociocultural contexts in which a technology is being developed is crucial to its design and deployment. The empirical investigations central to VSD methodology become particularly useful here in enrolling stakeholders and eliciting their values to ensure a more symbiotic mapping of values to design requirements (Fig. 2) and outcomes.

#### Value Identification

A starting list of values can be useful in determining a more cohesive and symbiotic set of values. Umbrello and van de Poel (2021) propose three sources of values:

1. Values that are to be promoted by the design (i.e., UN SDGs).
2. Values that should be respected, particularly those relevant to the AI: respect for human autonomy, prevention of harm (nonmaleficence), fairness and explicability (i.e., HLEG AI).
3. Values that are context specific and are not covered by (1) and (2) but which are derived from the analysis of a specific context in the first phase, in particular values held by stakeholders (e.g., emotional attachment in the context of care robot design).

It is during this phase that the values in consideration are interpreted and defined as per the VSD method of conceptual investigation. A normative approach to upholding these values throughout the design process becomes explicit.

#### Formulating Design Requirements

Using the proposed values of the previous subsection, we consider how these abstract values can be made into more concrete design requirements. The ‘values hierarchy’ (Fig. 2) is one method for translating this type of value-to-design requirement. The different sources of values are translated in different ways. The SDGs, for example, are to be considered throughout the design process as being *designed for* as much as possible, and provides a higher-level aim for design values. Whereas the HLEG AI principles are construed as boundary conditions or constraints that provide what can be understood as the minimum necessary conditions for acceptable design. Regarding the context for design, stakeholder elicitations and theoretical value lists are based on context and provide an important way for how any uncovered values are translated into design requirements. VSD has several established methods for undertaking this type of translation from stakeholder elicitations and other empirical investigations of stakeholder values. These methods include: value scenarios (Nathan et al., 2007), value sketches (Woelfer et al., 2011), value-oriented coding manual (Kahn Jr. et al., 2003), value hierarchies (Longo et al., 2020; van de Poel, 2013), value-oriented mock-up, prototype, or field deployment (Czeskis et al., 2010), value dams and flows (Denning et al., 2013), and value sensitive action-reflection model (Yoo et al., 2013).

#### Prototyping

Directly aligned with ‘value-oriented mock-up, prototype, or field deployment’ that Friedman and Hendry (2019) discuss, prototyping is the fourth stage where design requirements can be tested. More specifically, it is the

development, analysis, and co-design of mock-ups, prototypes and field deployments to scaffold the investigation of value implications of technologies that are yet to be built or widely adopted. Mock-ups, prototypes or field deployments emphasize implications for direct and indirect stakeholders, value tensions, and technology situated in human contexts.

(Friedman & Hendry, 2019, p. 62)

The first AI4SG ‘norm’ echoes this: “AI4SG designers should identify falsifiable requirements and test them in incremental steps from the lab to the “outside world”” (Floridi et al., 2020, p. 7). In other words, unforeseen or emergent values may come into play in the post-deployment stage, despite a system aligning with all the requisite design requirements, norms, and values pre-deployment (van de Poel, 2016). If such emergent factors do come into play during this step, another iteration of the four-stage cycle may be needed to integrate and align the design (Umbrello & van de Poel, 2021, p. 15).

### AI4SG Within a Care Context: PHAROS 2.0

In this section, we begin to integrate and further extend VSD within the AI4SG framework by situating it within the care domain. This is done by drawing on previous work on VSD within care practices, and more precisely, work on Care Centered VSD. In order to do that, we provide an example of a specific system that has recently been developed for the assistance of elderly people.

Physical Assistant Robot System (PHAROS) 2.0, is a socially assistive robot that monitors, evaluates, and advises older adults in performing physical activities and exercises at home (Martinez-Martin et al., 2019). This has been developed to help healthcare professionals evaluate the physical activity of elderly patients with illness outside of a hospital setting, and stimulate their overall health status (bodily and cognitively). PHAROS 2.0 can help by: providing exercise descriptions, both visually and verbally; recognising the type of exercise being done via visual input; giving feedback to the medical staff; and providing tailored recommendations to its users. All those tasks are accomplished with the use of machine learning techniques, such as Deep Learning (DL), which lets the system learn data features. Specifically, PHAROS 2.0 uses a combination of recurrent neural networks (RNN) and convolutional neural network (CNN) to identify and evaluate the physical exercises of users (Martinez-Martin et al., 2019, 2020). We examine the design of the PHAROS 2.0 prototype using the framework described above (i.e., Fig. 7).

### Context

As a tool for domiciliary care, the PHAROS 2.0 is designed to: relieve the burden on and to assist caregivers, homecare assistants, and healthcare professionals; promote a more comfortable treatment to elderly people outside of a hospital setting; and assist in the performance of activities for daily living (ADLs). Its deployment is also aligned with the promotion of active ageing—a combination of domains, e.g. familiar, social, professional,—that is designed to promote adequate responses to the needs of older adults and keep them active and engaged in social and individual activities (Paúl et al., 2017). For example, the NHS promotes active ageing and has provided a list of physical exercises that older people, or people with cognitive or physical disabilities, can perform at home.^2^ Design systems such as PHAROS 2.0 should aim to balance values tensions and remove the moral overload of *prima facie* conflicting values (van den Hoven et al., 2012). The prioritization of one value over another is strictly dependent on the context of use, which is either a hospital, a home, or a nursing home. For example, values of privacy and safety may be preferable to human contact and trust in domestic settings, since in such contexts the relation of trust between caregivers and care-receivers is already established and does not need to be prioritized (see also van Wynsberghe, 2013b). Therefore, the development of systems such as PHAROS 2.0 may assist in resolution of these kinds of value tensions in a way that still reduces health risks (bodily or cognitively) as much as possible.

### Value Identification

#### Values That Are to be Promoted by the Design, in Particular Deriving from the SDGs

The design of PHAROS 2.0 can be viewed as part of a larger network in support of #SDG 3, ‘Ensuring healthy lives and promoting well-being at all ages’. In particular, it may promote target #SDG 3.8: ‘the achievement of universal health coverage, including financial risk protection, access to quality essential health-care services, and access to safe, effective, quality and affordable essential medicines and vaccines for all’. Accessibility, accuracy, and affordability are central ethical values in the healthcare domain, which are of particular importance to designers, organizations, and industries that aim to develop systems that are driven by AI and machine learning. PHAROS 2.0 may help to create a more efficient and interconnected care network for individual caregivers and care-receivers, and for the healthcare infrastructure as a whole. Practically speaking, care robots can prevent unnecessary surgical interventions on vulnerable groups, and encourage meaningful and personalised care practices. These systems are beginning to develop beyond their original context to include care practices that require a social component. Although PHAROS 2.0 has already been deployed, to meet with new design requirements it will be integrated with social interaction skills in the near future (Martinez-Martin et al. 2019, p. 4).

#### Values That Should be Respected, in Particular Those Values That Have Been Identified in Relation to AI: Respect for Human Autonomy, Prevention of Harm (Nonmaleficence), Fairness and Explicability

This second source value is values that are to be promoted in AI.

##### Respect for Human Autonomy

We are increasingly interacting with systems that are imbued with autonomous decision-making in different domains. These systems influence our lives in various ways, from shaping the context in which the individual makes a decision, to altering interactions between individuals, and assumptions of democratic participation. Human autonomy in this context is the balance between an agent’s retaining as much freedom of choice as possible and the delegation of decision-making to systems. Systems in turn should be designed in such a way as to promote autonomy, to avoid cases in which a systems’ efficacy falls short in making consistent and coherent decisions on the behalf of its human users (Floridi et al., 2018). Regarding care practices in CCVSD, the degree of a system’s autonomy can be evaluated through using three of van Wynsberghe’s four fundamental values of care: attentiveness, competence and reciprocity in the tasks. Human autonomy is an important consideration in what van Wynsberghe calls systems’ ‘appearance of moral agency’ (van Wynsberghe, 2016, p. 313). In other words, because systems are used in inherently ethical contexts i.e. being responsible for vulnerable groups, such as elderly people, this could compromise human autonomy. Thus, the ‘appearance of moral agency’ in the PHAROS 2.0 should be sustained and advanced by a contextual analysis of the care practices at stake and, with deference to the respective personal choices of caregivers in their assistance, and care-receivers’ in their treatments. For example, the designers of PHAROS 2.0 have proposed a new version of its recommendation strategy to meaningfully engage its users in the active ageing process. In fact, systems that are designed to give personal recommendations have enriched information provided by the user, which results in a more tailored workout that has a batch of exercises instead of just one (Martinez-Martin et al., 2019, pp. 8–13).

##### Prevention of Harm (Nonmaleficence)

This value seeks to avoid harm, and the risk of harm, by understanding systems’ capability and limitations. In the case of PHAROS 2.0, harm may occur due to the way the system has been designed for users. By promoting the well-being and safety of its users, the system risks valuing this over the users’ need for human contact and autonomy. The PHAROS 2.0 and other similar systems have raised privacy concerns because of their access to a users’ personal information. Systems observe and record care-receivers in order to provide effective care, but this has led to concern about the data practices of storing, archiving, collecting data, and monitoring care-receivers. PHAROS 2.0 has several databases with users’ personal information, including their health condition, exercise information, and their caregivers’ information (Martinez-Martin et al., 2019, p. 9). This data collection potentially infringes upon the privacy of care-receivers and the healthcare system more broadly, risking an exposure of privileged information to being shared with third parties. In terms of the design of AI-driven systems what can be discussed and problematized is the quality of the training data and the reliability of the algorithms used for predictions, such as the one used for recommendation in PHAROS 2.0.

Compared to the CC Framework, nonmaleficence can be subsumed under the value of competence, which assesses systems’ capability and limitations in executing a task. These systems’ capabilities may include: safety, efficiency, quality of task execution, force feedback, tactile perception, and other capabilities (van Wynsberghe, 2012, p. 111). Nevertheless, values such as efficiency and privacy are obscured in CCVSD, because they are considered as exclusively driven by either consequences or duties, and lack the ability to take into consideration the overall development of users’ ethical character (van Wynsberghe, 2016). However, we can arguably include these values in the list of moral elements, as they gain increasing relevance in the design and deployment of AI-driven systems.

##### Fairness

According to Floridi et al. (2018), the value of fairness can be framed as justice and can be defined in a tripartite way: (1) Using AI to correct past wrongs such as eliminating unfair discrimination; (2) Ensuring that the use of AI creates benefits that are shared (or at least sharable); (3) Preventing the creation of new harms, such as the undermining of existing social structures. The value of fairness in care practices refer to the allocation of healthcare resources and services on the basis of objective and fair health related needs and factors. This should also include the values of accessibility and affordability, which are strictly aligned to SDG #3 as noted above (5.2.1).

##### Explicability

This means that AI systems should be intelligible and transparent, and there should be at least one human agent that can be considered accountable for how the system works (Floridi et al., 2018). In van Wynsberghe’s works explicability is not explicitly mentioned. However explicability may be linked to the notion of trust, which she writes is a “hybrid event between the human caregiver and the robot” (van Wynsberghe, 2013a, p. 428). Trust is closely aligned with the value of responsibility (van Wynsberghe, 2012, 2016) insofar as they relate to the capacity to be held accountable and liable, but the idea of trust is never formally introduced or systematically used in the discussion of moral elements in the CC framework. PHAROS 2.0 replaces homecare assistants or specialised instructors in the monitoring and advising of older adults. In the near future it may also replace those actors in providing the elderly with company and helping them in emergency situations. The delegation of tasks to these systems may lead to other scenarios such as the disappearance of certain types of medical and caregiving professions, and the reallocation of expertise and responsibilities in healthcare systems.

Finally, such systems replace caregivers and can introduce new forms of attentiveness and competence that may lead, over time, to the establishment of trusting bonds. In this scenario, trust is not a bond between two actors interacting, but between multiple actors. These include the healthcare systems, the healthcare staff that are assisted, the third-party providers that implement the systems, and the institutions and policy makers that regulate the systems’ introduction and use. To further explore trust and its link with explicability, the focus of our approach should not be on the reciprocal engagement of the robot compared to the human, but on how the “forum” (van Wynsberghe, 2016) of trust has changed from being associated with the traditional relationship between caregivers and care-receivers to a new and unprecedented one.

#### Context-Specific Values That Are not Covered by (5.2.1) and (5.2.2) by Which Derive from the Analysis of the Specific Context in Phase, in Particular Values Held by Stakeholders

We have shown how the PHAROS 2.0 system has been developed in response to the ever-increasing elderly population and the need to promote active aging via a more personalised approach to the elderly population’s overall health status (bodily and cognitively). Many of the values and side effects of the PHAROS 2.0’s deployment have been discussed, such as the values of *general health and well-being* (under 5.2.1) and *autonomy*, *non-maleficence, fairness as justice,* and *explicability* (under 5.2.2). For example, PHAROS 2.0 may give a user a false sense of security with regard to their general health or well-being, or it may increase the users’ dependency on technological systems to the detriment of their autonomy. It may also lead to unintended discriminations due to the systems’ potential lack of accessibility and affordability, and thus its fairness, in its dissemination to potential users. However, other values can be less clearly subsumed under the two source values outlined above. A contextual analysis serves to consider such classes of values, which are related to stakeholders’ values and preferences (see 5.1).

One of the possible values at play in the context-specific level may be emotional attachment, which is strictly aligned with trust. Current robots are said to be incapable of giving the ‘‘real compassion and empathy or understanding’’ that is found in human companionship (Sharkey, 2014). From a CCVSD perspective systems such as PHAROS 2.0 should be designed in a way that promotes the value of responsiveness or reciprocity (van Wynsberghe, 2016) to encourage human autonomy and, potentially, the foundation to build a bond of trust between the care-receiver and the care robot. Therefore, at this stage, VSD methodological tools such as envisioning cards (Friedman & Hendry, 2012), which are designed to evoke considerations and discussion, may help in reconstructing stakeholders’ values and in the modelling of physical human–robot interactions in response to users’ preferences. It is of crucial importance also for the future development of care practices and the long-term and indirect effects that context-specific values are thoroughly understood, and then translated into design requirements.

### Formulating Design Requirements

**Fig. 8:**
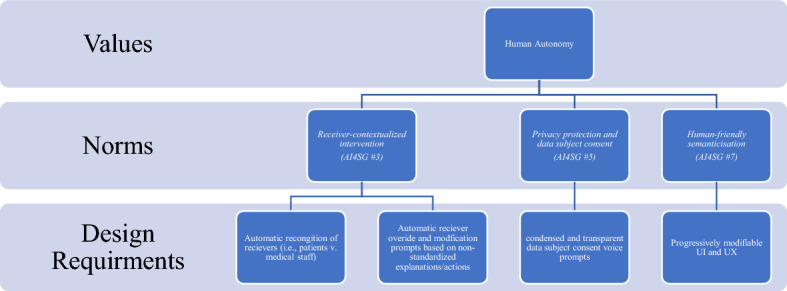
Translating human autonomy to design requirements through AI4SG norms

There is a variety of instruments and methods in the VSD methodology that can be adopted to help designers actualize necessary design requirements into any given design. As we have demonstrated, the ‘values hierarchy’ (Fig. 2) is particularly useful as a way to illustrate and trace design requirements from norms and value, and vice versa. Figure 8 is one example of how to visualize the translation of higher-level values, through AI4SG norms and into technical design requirements.

**Fig. 9:**
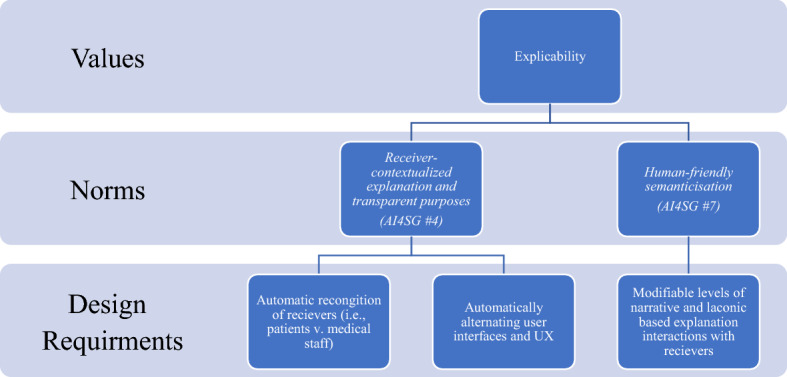
Translating explicability to design requirements through AI4SG norms

Figure 8 visualizes the abstract value of *respect for human autonomy*. It is translated through various AI4SG norms (3, 5, and 7), which are illustrated in Fig. 6, into more concrete design requirements. Umbrello and van de Poel (2021) construe the AI4SG factors as norms, rather than as abstract values in design in light of recent work by Floridi et al. (2020) which describes norms as being imperatives for designers. It should be noted that the context of use will naturally change any given combination of values, norms and subsequent design requirements. Figure 8 is just one illustration of how this process, and given our earlier example of the PHAROS 2.0, the value of *respect for human autonomy* is not necessarily the predominant value in a design. To this end, “there is no exclusive nor exhaustive route for satisfying a value translation” (Umbrello & van de Poel, 2021, p. 19). Both *prevention of harm* and *explicability* for example overlap with *respect for human autonomy*, given that they implicate AI4SG norms 5 and 7 respectively (see Fig. 9). As a result, these values mutually co-vary and, in many cases, should be used to operationalize each other. Design requirements that are translated from the value of *explicability,* for example, can be used as a route for engaging with and operationalizing the value of *situational fairness* and *prevention of harm*.

Functionally speaking, the AI4SG norms are apt at avoiding *most* of the harms that may emerge as a consequence of AI systems deployment, but not all. However, this does not mean that such systems will necessarily positively contribute to social good. “Global beneficence” that is, contributing to global good, is an inherent part of engaging with higher-level values like the *actual* operationalization of the SDGs as discussed above. For this reason, our paper has adopted this multi-tiered approach to VSD. By combining values specific to AI and stakeholder values, with due consideration of SDG targets, as met by AI4SG norms, concerns around the sanctioning of AI systems that do not respect these central ethical principles can be mitigated.

### Prototyping

Prototyping requires building mock-ups based on design requirements (see Sect. 4.4.4). In doing so, the technologies move out of the conceptual space to become imbibed with the values of the stakeholders. Widespread testing type activities take place to determine if any of the design decisions prove to be intractable in practice that were not during their development in the design space. This is also the stage that determines if there are any emerging technical or ethical issues that were not foreseen at previous stages of the VSD methodology (van de Poel, 2020). Because care robots are in limited deployment rather than ubiquitous rollout, these systems retain the ability to be recalled from operation and brought back into the design sphere. This means that more iterations of the VSD approach can be undertaken to account for any unforeseen issues that might emerge post-deployment. Unlike the SARS-CoV-2 contact-tracing app which has obvious incentives for immediate deployment (Umbrello & van de Poel, 2021), the development and deployment of the PHAROS 2.0 robot is not motivated by similar immediate need. Technologies like PHAROS 2.0 have the advantage of small scale testing and direct stakeholder mock-ups, which means that they are more capable of affirming design values and of avoiding the harms that would have inevitably emerged if it were deployed as part of an emergency rollout.

Prototyping is not strictly a lab-driven activity that is focused solely on testing technical aspects in a decontextualized setting. On the contrary, a crucial part of the prototype stage is to observe and evaluate the social and ethical effects of a design from its limited deployment in the field. Prototyping is a form of interactional design that draws on a participatory design approach where designers and stakeholders work together in an organisational or context-related setting like that described by Bødker (2015). The PHAROS 2.0 robot is a particularly apt case. Values such as *respect for human autonomy* can be affirmed through various design decisions, such as integrating an automatic receiver recognition, which allows PHAROS 2.0 devices to communicate with multiple agents such as medical staff, homecare professionals, and patients. On the other hand, other values such as *situational fairness* may require of the moment insights with stakeholders to understand the more nuanced behaviors that affect the salient design of more abstract values. For this reason, it may be more effectual to begin with and gradually scale up small-scale mock-ups to ensure that the progressive iterations of the methodology sufficiently account for the changing and emerging values in each deployment and are accounted for in the design (c.f., van de Poel, 2020). This type of approach can aid designers to discover new values, prompting another iteration of the cycle that might not have been triggered otherwise.

## Conclusions

The values involved in the design of care robots are present across various levels of abstraction. This paper began by introducing the values that are central to care in the design of care robots for the elderly. In particular, we used the Care-Centered Value Sensitive Design (CCVSD) approach as a starting point for robotics and care ethics. We expanded on this by presenting other sources of values such as the UN SDGs, higher-level norms to be promoted by design (as much as possible), the European Commission’s HLEG AI values of beneficial AI as constraining values to be designed for (as much as possible), and finally contextual values, in particular those stemming from stakeholder elicitations. These three sources of values were then translated into design requirements via norms that are specific to AI systems, i.e., AI4SG factors. In doing so, we adopted the value sensitive design (VSD) approach and modify it according to Umbrello and van de Poel’s (2021) framework for a multi-tiered approach to the ethical design of AI systems. Drawing on the example of the PHAROS 2.0 socially assistive robot, this paper has provided an experimental approach forhow designers can begin to direct their practices towards designing AI *for* social good.

## Footnotes

1 Department of Health and Social care. NHS health information available through Amazon’s Alexa. 2019. Available from: https://www.gov.uk/government/news/nhs-health-information-available-through-amazon-salexa. Accessed March 19, 2021.

2 NHS Choices. Exercises for Older People. 2018. Available from: https://www.nhs.uk/Tools/Documents/NHS_ExercisesForOlderPeople.pdf. Accessed on March 19, 2021.
